# Animal Models of LED-Induced Phototoxicity. Short- and Long-Term In Vivo and Ex Vivo Retinal Alterations

**DOI:** 10.3390/life11111137

**Published:** 2021-10-26

**Authors:** Juan A. Miralles de Imperial-Ollero, Alejandro Gallego-Ortega, Arturo Ortín-Martínez, María Paz Villegas-Pérez, Francisco J. Valiente-Soriano, Manuel Vidal-Sanz

**Affiliations:** 1 Departamento de Oftalmología, Universidad de Murcia e Instituto Murciano de Investigación Biosanitaria (IMIB) Virgen de la Arrixaca, Campus de CC de la Salud, El Palmar, 30120 Murcia, Spain; juanantonio.miralles@um.es (J.A.M.d.I.-O.); alejandrogallego@um.es (A.G.-O.); mpville@um.es (M.P.V.-P.); 2 Donald K. Johnson Eye Institute, Krembil Research Institute, University Health Network, Toronto, ON M5T 2S8, Canada; Arturo.OrtinMartinez@uhnresearch.ca

**Keywords:** LED induced phototoxicity, cone photoreceptor, microglia activation, retinal pigment epithelium, neuroprotection

## Abstract

Phototoxicity animal models have been largely studied due to their degenerative communalities with human pathologies, e.g., age-related macular degeneration (AMD). Studies have documented not only the effects of white light exposure, but also other wavelengths using LEDs, such as blue or green light. Recently, a blue LED-induced phototoxicity (LIP) model has been developed that causes focal damage in the outer layers of the superior-temporal region of the retina in rodents. In vivo studies described a progressive reduction in retinal thickness that affected the most extensively the photoreceptor layer. Functionally, a transient reduction in a- and b-wave amplitude of the ERG response was observed. Ex vivo studies showed a progressive reduction of cones and an involvement of retinal pigment epithelium cells in the area of the lesion and, in parallel, an activation of microglial cells that perfectly circumscribe the damage in the outer retinal layer. The use of neuroprotective strategies such as intravitreal administration of trophic factors, e.g., basic fibroblast growth factor (bFGF), brain-derived neurotrophic factor (BDNF), ciliary neurotrophic factor (CNTF) or pigment epithelium-derived factor (PEDF) and topical administration of the selective alpha-2 agonist (Brimonidine) have demonstrated to increase the survival of the cone population after LIP.

## 1. Introduction

### 1.1. Age Related Macular Degeneration

Age-related macular degeneration (AMD) is a chronic, progressive degeneration of the retina that causes loss of central vision in people over 50 years of age [1,2]. At present, AMD is the leading cause of blindness in the elderly in developed countries [3]. AMD is a common disorder affecting 8.7% of the population aged 50–85 years worldwide [4].

AMD is characterised by degeneration of the outer retinal layers: photoreceptors, Bruch’s membrane and retinal pigment epithelium (RPE) in the macula [5]. Its cause remains unknown, although numerous risk factors have been identified [2]. Age, genetic factors (CFH, ARMS2), Caucasian race, tobacco smoking, obesity or high fat diet are well known risk factors [6,7,8,9]. Others like diabetes mellitus and high blood pressure are discussed risk factors [10,11]. Environmental conditions and in particular exposure to sunlight, more specifically blue or short wavelength light may predispose to the onset of the disease [2,12,13,14]. Animal research has shown that chronic exposure to blue or ultraviolet light is harmful to photoreceptors and the RPE [15,16]. Although clinical studies have documented the deleterious effects of acute exposure to light on the retina [17], the effects of chronic exposure to light remain to be shown. Thus, different animal models of phototoxicity have been developed to study light-induced retinal degeneration in order to better understand AMD and prevent its onset [11,18,19].

### 1.2. Phototoxicity Models

Phototoxicity is defined as the adverse effect of exposure to light on tissues due to its absorption. The first published work documenting the effect of phototoxicity on the rodent (rat) retina was done by Noell et al. (1966) [20]. Since then, the interest of researchers on retinal phototoxicity has been increased and numerous phototoxicity models have been developed. These models can be classified according to: (i) in vitro or in vivo studies [21,22,23,24,25,26,27,28,29,30,31]; (ii) wavelength of light: although white light have been the most studied by researchers [23,26,32,33,34,35], other light spectra such as blue light (400–470 nm) [22,23,24,26,36,37,38,39,40,41,42,43,44] or green light (507–535 nm) [22,23,31,45] have also been studied; (iii) light source: fluorescent sources [33,34,35,41,46,47,48] or LED sources [22,23,26,32,36,37,38,39,42,43,49,50]; (iv) intensity and duration of photo-exposure: focal models require short exposure times [37,38,39,40,44,50] while the diffuse models require longer exposure times [22,23,26,33,34,36,41,42,43,46,47].

### 1.3. LED Phototoxicity Models

Although many phototoxicity models use white light due to its similarity to sunlight, the increasing use of LEDs [21,22,23,24,26,35] in recent years there has been a growing interest in the study of the effect of blue light on the retina and the RPE by developing many diffuse phototoxicity models [22,23,29,36,37,39,40,41,42,43,51,52,53].

#### 1.3.1. Diffuse LED Phototoxicity Models

The use of LEDs as lighting sources has become increasingly popular over the years in homes and cities due to the reduced cost and the quality of light they provide. In addition to the economic and environmental value, these devices can provide not only white light but can be modulated to emit any wavelength without the need for light filters, and studies of the effect on the retina of different lights, depending on their wavelength, have proliferated in recent years. Table 1 shows the experimental models using diffuse exposure, whose light source is provided from several LEDs placed around the cages, produce a diffuse retinal injury documented by many authors as a decrease of the outer retinal thickness [23,24,36,41] and functionally by a decrease of the a-wave and b-wave in the electroretinogram [36,41]. Most studies show the retinal alterations produced by exposure to white or blue light, although authors such as Jaadane et al. (2015) [22] or Krigel et al. (2016) [23] have also documented the damaging effect of green light. The LED exposure time is highly variable between studies, ranging from constant exposures of 2 to 72 h [22,23,27,32,36,42,43] to cyclic exposures of 3 to 12 h per day [23,24,26] (Table 1). Light intensity has also varied from 150 lux [24] in Brown-Norway rats to 6000 lux [23] in Wistar and Long Evans rats. Of the studies reviewed, most use albino rats or mice and only two use pigmented ones [23,43]. Krigel et al. (2016) [23] show that under the same LED phototoxicity conditions, the retinas of albino rats are more affected than pigmented rats. This can be seen in the studies of Nakamura et al. (2017 and 2018) [42,43] who documented that albino mice need to be exposed to blue LED light at 400–800 lux for 2 h and pigmented mice at 1100 lux for 3 h to obtain similar retinal phototoxic damage. Therefore, albino animals are more sensitive than pigmented animals to LED phototoxic injury. The periods of analysis of the effects of light on the retina also vary, with many studying the effects just after the induction of phototoxic damage [22,23,24,26,32] and others studying the effects over a longer period of time (7 days) [23,43].

#### 1.3.2. Focal LED Phototoxicity Models

Ortín-Martínez et al. (2014) [37] developed a focal model of blue LED-induced phototoxicity (LIP) in albino rats in order to generate a focal phototoxic retinal damage. Later, other authors from the same laboratory have further studied the long-term effects in albino rats [50], applied this model albino mice [38,39] and, more recently, in pigmented mice [40]. The aim of these experiments was to develop a reliable and reproducible blue LED-induced phototoxic injury model. This model produces a lesion in the superior-temporal region of the retina, the region with the highest density of cones and RGCs in rat and mouse retinas [55,56]. In this model, light passes through the cornea and lens before reaching the retina. The transmittance for blue light (400 nm) through these rat ocular tissues is ≈78% [57]. Therefore, it is assumed that in this model approximately 80% of the light emitted by the diode reaches the retina of the rodent.

### 1.4. Distribution of Cone Population in Rats and Mice

Previous works had identified, counted and mapped the entire population and distribution of S- and L-opsin^+^ cones in albino and pigmented rats [55] and mice [56] using automated computerised routines. The retinas of rats and mice contain only two types of cones, S and L/M (L). 

The distribution of S-cones in albino and pigmented rats is mainly concentrated at the retinal borders with an increasing gradient in the inferonasal quadrant of the retina [55] and L-cones show a more homogeneous distribution throughout the retina, with a higher density in the central retina and, specifically, in the superior-temporal quadrant [55] where the highest density of retinal ganglion cells (RGCs) is also found [58,59], so this region is known as the “visual stria” and would emulate the human fovea [55]. In addition, the percentage of genuine S- or L-cones in the whole mounted retinas was 13.6% and 83% respectively, in albino rats and 9% and 89% respectively, in pigmented rats [55]. The presence of dual cones (co-expression of S- and L-opsin) in both rat strains was approximately 3% [55]. 

In the mouse retina, S- and L-cones are distributed throughout the retina differently than in the rat. Moreover, their distribution depends on the mouse strain. In the albino mouse, S-cones are homogeneously distributed throughout the retina, with a predominance in the ventral retina [56]. However, in pigmented mice they are concentrated in the inferonasal quadrant, while the superior-temporal region is devoid of S-cones [56]. In contrast, L-cones are homogeneously distributed throughout the retina in both strains [56]. In terms of opsin expression, pigmented mice had significantly more total cones and L-cones and 40% of the cones were dual cones whereas in albino mice the percentage of dual cones was 76% and, immunodetection of S-opsin detected 96% of the total number of cones [56]. Recently, arrestin^+^ cones have been counted and mapped in pigmented mouse whole-mount retinas identifying a higher density in the central retina than in the periphery and a 99% co-expression with L-cones and 92% with S-cones [40].

## 2. Toxic Effects of Focal LIP on the Retina

Although light is necessary for the vision, the energy of light can also damage the retinal cells. The light energy absorbed by any tissue depends on its absorption of light [60]. It has been documented that light exposure can damage photoreceptor and RPE cells [40,43]. The deleterious effect of light is classified depending on the type of damage induced [16,60]: (i) photomechanical damage: produced by rapid inputs of light which cause compressive or tensile forces and lead to microbubbles formation and the destruction of RPE and other cells; (ii) photothermal damage: photons of light can be absorbed by retinal tissue, specially by the melanosomes of the RPE and melanocytes of the choroid, and produce the denaturation of molecules and formation of abnormal molecular linkage which lead to the loss of function of the cells; (iii) photochemical damage: light energy excites the electron of different molecules of the retina. The return of this electron to baseline releases the extra energy producing electron exchange between molecules and the formation of reactive oxygen species (ROS). 

The physiopathology of photoreceptor death has been studied in many models of phototoxicity and three possible hypotheses have been proposed by Noell et al. (1966) [20]. The first hypothesis for the photoreceptor degeneration would be through an alteration of the vitamin A caused by light. In the second hypothesis, light would produce a metabolic alteration in the photoreceptor that would lead to its degeneration and the third hypothesis would involve oxidative reactions as the cause of the deleterious effect of light on the photoreceptor. To date, although not all details of the pathophysiology of light on the retina are known, it is known that phototoxicity is a multifactorial process involving genetic and environmental factors in which programmed death or apoptosis plays a major role [22,23,61], although the presence of necrosis has also been suggested [22,23]. Light, especially the blue spectrum, increases ROS, which contribute to photoreceptor degeneration through lipid peroxidation and cell apoptosis [24,26,30,36,52,62,63,64,65,66].

### 2.1. Retinal Thickness Reduction and Cone Degeneration in Models of LED-Induced Focal Photoreceptor Phototoxicity

Retinal degeneration in rats and mice photo-exposed to blue LED in a focal phototoxicity model has been studied in vivo by spectral domain optical coherence tomography (SD-OCT) [37,39,40] and electroretinography (ERG) [39] and ex vivo by immunohistochemistry [37,38,39,40]. 

The protocol of the induction of light-induced retinal damage was similar between rats and mice. Prior to photo-exposure, rodents were dark-adapted for at least 12 h and subsequent manipulation was performed under long wavelength red light (λ > 600 nm), to which rats and mice are not susceptible [67,68]. After pupil mydriasis, the left eyes were photo-exposed to a blue LED (emission spectrum 390–410 nm; catalogue number 454–4405; Kingbright Elec. Co., Taipei, Taiwan) connected to a computer to control the time and intensity of exposure (10–20 s and 200 lux for albino rodents and 45 s and 500 lux in pigmented mice) [37,38,39,40]. The diode was held with a micromanipulator and placed 1–2 mm perpendicular to the corneal apex of the left eye. In these focal photo-exposure models, the light converged on a specific area of the retina and produced a focal lesion in the superior-temporal quadrant of the rodent retinas. This lesion could be visualised and monitored in vivo using SD-OCT [37,39,40]. 

#### 2.1.1. In Vivo SD-OCT Observations in the Focal LIP

In albino rats [37,50], retinal damage was confined to a circular area measuring approximately 1.8 mm in diameter at 12–24 h after LIP and slightly decreasing progressively at 7 days after LIP. Optical sections acquired with SD-OCT in the center of the lesion showed progressive retinal thinning that decreased by approximately 40% at 7 days post-LIP. In addition, a hyper-refringent reaction was observed in the first days of the study, progressively disappearing, and leading to retinal atrophy; however, only the total retinal thickness was measured, and no differentiation was made between outer and inner retina.

In 2019, Valiente-Soriano et al. [39] adapted the focal phototoxicity model to albino mice with the same exposure time and intensity previously used in albino rats (10 s and 200 lux). SD-OCT “en face” showed a circular lesion and SD-OCT section scans showed degeneration events in the outer retina very similar to those documented in albino rats. In particular, there was a hyperreflective region in the outer retina whose origin is unknown to the authors, although different hypotheses have been developed: restructuring of the outer layers, cellular detritus, microglial mobilisation or retinal oedema after light damage. Quantitative analysis showed that total retinal thickness in the center of the lesion decreased by approximately 33% at 7 days after LIP compared to control retinas. This work also incorporated a longitudinally in vivo full field electroretinographic study in which retinal functionality was analysed at days 1, 3 and 7 after LIP. The recordings showed a significant reduction in the amplitude of the a- and b-wave response one day after LIP in photo-exposed eyes that recovered at 3 days and was maintained at 7 days. Therefore, it is likely that the small LIP-induced focal lesion affecting the cones, and most likely also the rods, is not sufficient to make these ERG a- and b-wave alterations permanent over time [39].

Recently, the focal phototoxicity model has been adapted to pigmented mice by increasing the time and intensity of exposure to 45 s and 500 lux [40], in accordance with previous studies in pigmented rodents [23,43]. In vivo analysis of photo-exposed retinas showed the focal lesion in the superior-temporal retina and that total retinal thickness in the center of the lesion was reduced by 35% at 7 days post-LIP, a similar loss to that previously shown in the albino rat [37] and mouse [39]. The authors showed that the loss of retinal thickness was entirely due to the outer retina, which was drastically reduced by 68% within 7 days of LED exposure.

#### 2.1.2. Ex Vivo Histological Observations in Focal LIP

In albino rats, this focal lesion was also visualised and analysed ex vivo by immunohistochemistry, approximately 3.4 mm from the optic nerve. S- and L-cone populations were immunodetected in the total retina and quantified following computer routines previously developed in the same laboratory [55,56]. Fluorescence microscopy revealed the focality of the lesion in this model, affecting only the superior-temporal retinal region. However, automated counting of whole retinas showed no significant differences between experimental and contralateral uninjured retinas. Retinal analysis showed that S-cones were more sensitive to blue light than L-cones, with a significantly larger area of lesion. Therefore, the cone populations were studied and quantified within a prefixed circular area (PCA) of 1.3 or 1 mm of radius to count S-cones or L-cones, respectively, centred on the lesion. Analysis of cones within the PCA after LIP revealed a 73 and 40% decrease of S- and L-cones at 7 days after LIP and remained constant at 30 days after LIP. Thus, S-cones were reduced by a higher percentage than L-cones and therefore showed a higher sensitivity to blue light [37].

For the ex vivo study in the albino mice, the authors used S-opsin as a cone marker due to the high percentage of dual cones in the albino mouse retina, which allows the detection of approximately 96% of cones with S-opsin expression and that are homogeneously located throughout the retina [56]. OS degeneration was studied in a 0.8 mm diameter PCA placed in the centre of the lesion, located approximately 1.2 mm from the ON, showing a significant reduction of 58% of the S-cone population at 7 days [39].

In the pigmented mice, the ex vivo study showed that the lesion was located approximately 1 mm from the ON with an area of involvement of approximately 0.25 ± 0.02 mm^2^, which is 1.6% of the total area of the mouse retina [40]. Thus, cone degeneration analysis was performed on the area of damage within a 0.9 mm diameter PCA and showed a significant reduction of arrestin^+^ cones at 3 days after LIP that progressed to 7 days later, when cones within the PCA decreased by 35%. These data are similar to the previously mentioned L-cone data for albino rats, which reflected a 40% loss [37] and significantly less than the S-cone loss documented in albino rat [37] and mouse [39], which reflected a loss at 7 days after LIP of 58% and 73%, respectively. These data show that in this model of LED phototoxicity using blue light, the S-cones are more sensitive to exposure, probably due to the deleterious effect of scattered light on these photoreceptors whose opsin has a maximum absorption peak at 450 nm [37].

To study apoptotic cell death in blue light LED-induced focal photo-exposure in photoreceptor degeneration, a TUNNEL assay, which detect fragmented and damaged DNA to identify cells undergoing apoptosis in a late stage, was performed in retinas of albino mice after LIP. TUNNEL-positive nuclei in the ONL peaked at 3 days after LIP and progressively decreased at 7 days. TUNNEL-positive nuclei were detected only in the lesion area and in the ONL which peaked at 3 days after LIP and progressively decreased at 7 days, demonstrating the involvement of apoptosis in cone degeneration in this model.

### 2.2. Microglial Reaction in Focal Phototoxicity Models

Described in 1939 by Pío Del Río-Hortega [69], microglial cells are part of the innate immune system resident in the CNS and retina. These cells reach the retina during embryonic and postnatal development by migrating from the blood vessels of the ciliary body, optic nerve and hyaloid vessels [70,71,72]. These cells have been extensively studied in recent years for their involvement in retinal degenerative processes as their main function is to monitor the retinal microenvironment to detect stimuli or injuries that damage retinal cells [70,72]. Originally, the function of these cells was thought to be reduced to that of a simple spectator of disease, responsible for removing cellular detritus and degenerated cells [73]. However, in recent years, the important role of microglia in systemic neurodegenerative diseases such as Multiple Sclerosis, Amyotrophic Lateral Sclerosis, Alzheimer’s disease and Parkinson’s disease [73,74,75,76], and in other ocular pathologies such as photoreceptor degenerations, has been characterised [77] and have been shown to play a key role in modulating disease and in the ability to prevent or slow cell death progression. 

One of the most important aspects of the study of retinal microglia is their morphology, which has been well described according to the retinal layers in which they are located in both basal and pathological conditions [78,79,80]. In intact rat or mouse retinas, microglial cells are found in several retinal layers with a characteristic relaxed-branched morphology. Most of them reside in the layer of RGCs, IPL and the OPL, with little or no presence in the outer segment layer (OSL) [78,79,81]. Several authors have described the unique ability of microglia to activate and migrate to a specific area of injury, reaching the retina from other parts of the central nervous system, referring to this phenomenon as the spider effect [82,83,84,85]. In models of diffuse LED induced phototoxicity, a recruitment and activation of microglial cells in the outer retinal layers has been described in the whole retina following light damage that resembles observations in human tissue specimens of AMD [86,87]. 

**Figure 1:**
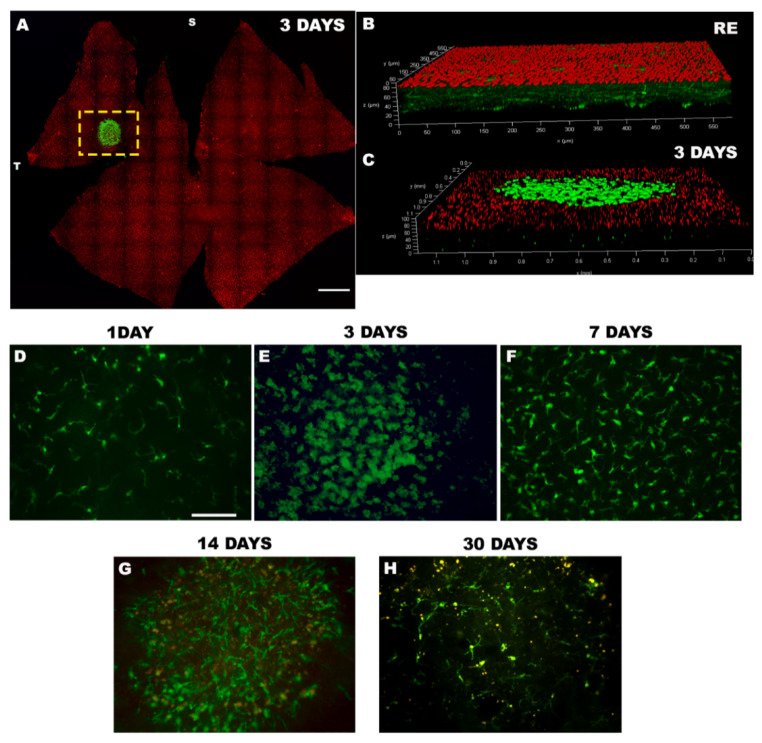
Iba-1^+^ cells in outer segment layer (OSL) after LIP. Wholemount retina of a retina photo-exposed 3 days after LIP with an Iba-1^+^ reaction in the OSL after LIP (green) in the centre of the lesion in an albino rat retina (**A**). Confocal magnification of a right eye retina (RE) without microglial cells in OSL (red; **B**) and of a damage retina at 3 days after LIP where Iba-1^+^ cells (green) were observed in the center of the lesion and localised in OSL (red; **C**). Magnifications of Iba-1^+^ cells located in OSL at 1, 3, 7, 14 and 30 days after LIP in which morphological changes were observed from a dendritic shape at 1 day to an ameboid shape at 3 days after LIP and progressively returning to an elongated morphology (**D**–**H**). Bar scale in A = 1 mm; in D-H = 50 µm.

The reaction of microglial cells has been investigated in the focal LIP model. Alterations in microglial cells have been observed within the circular area of the lesion, [39,40] which is delimited in its extent in the superior-temporal quadrant of the retina as shown in Figure 1A,C corresponding to an albino rat retina analysed 3 days after LIP. The evolution of the appearance and morphology of Iba-1^+^ cells in the lesion area has been also studied, both in the short and long term after induction of phototoxicity. The process of activation and migration of Iba-1^+^ cells is similar between albino rats [37] and albino [39] and pigmented [40] mice within hours of LIP induction within the phototoxic lesion area. All these studies agree that the highest number of Iba-1^+^ cells in the OSL occurred 3 days after LIP when the number of OS within the PCA had significantly decreased compared to control retinas (Figure 1A–C). However, as shown in Figure 1D, activation of these cells in the OSL is already beginning to be observed 24 h after LIP, although these cells still maintain a relaxed-branched shape with long primary processes and numerous secondary processes born from an elongated soma. At 3 days after LIP, the activation of these cells is complete and they perfectly circumscribe the lesioned area, acquiring a round-amoeboid morphology shape with a large soma with vesicles inside, from which emerged numerous and shorts processes (Figure 1A,C,E). At 7 days post-LIP the shape of the cells progressively changed to a dendritic (less active) morphology with longer processes and smaller somas with more amoeboid cells at the periphery of the lesion (Figure 1F). These more relaxed-branched morphologies were more evident at 14 and 30 days when, in addition, a rounded autofluorescent deposit was observed, detectable with different filters, mainly at the periphery of the lesion, both inside and outside the cells, which showed its autofluorescent nature (yellow signal on panels G and H in Figure 1) and whose origin was probably cellular detritus. Finally, 30 days after LIP, the presence of Iba-1^+^ cells in OSL at the lesion site was less obvious.

The origin of the migration and activation of these cells is debated in the scientific community. As mentioned above, the Iba-1^+^ cells described 3 days after LIP had a very rounded morphology with very short and thin processes. However, some authors have described these cells as highly activated microglial cells [85,88] while other authors have found cells with similar morphological characteristics in mice after OHT located in the retinal nerve fibre layer (RNFL) and in the RGC layer suggesting that the nature of these cells may be that of macrophages extravasated from the bloodstream by a rupture of the inner blood-retinal barrier (BRB) [78,79]. Similar findings have also been observed in models of diffuse phototoxicity in which blood-derived macrophages were identified in the outer retinal layers together with retinal resident microglia after retinal photo-exposure. Similar findings have also been observed in models of diffuse phototoxicity in which blood-derived macrophages were identified in the outer retinal layers together with retinal resident microglia following retinal photo-exposure via the ON and the ciliary body as a route of entry for these macrophages [86]. The involvement of exogenous macrophages is very difficult to determine in focal phototoxic injury by immunohistochemical techniques, so although it is possible that the origin of the amoeboid cells is microglial activation, it cannot be ruled out that part of this presence is due to macrophage extravasation from the bloodstream due to an alteration of the BRB.

Microglia were also studied in the outer plexiform layer (OPL) in pigmented mice. Focal LED photo-exposure did not change the number of microglial cells in the lesion area (within the OPL) compared to homologous and naïve retinas, which may be attributed to the small lesion produced by this model [40]. This indicates that the activity of these Iba-1^+^ cells is focused only on the affected retinal layer without interfering with the other layers.

### 2.3. RPE Degeneration in a Focal Phototoxicity Model

**Figure 2:**
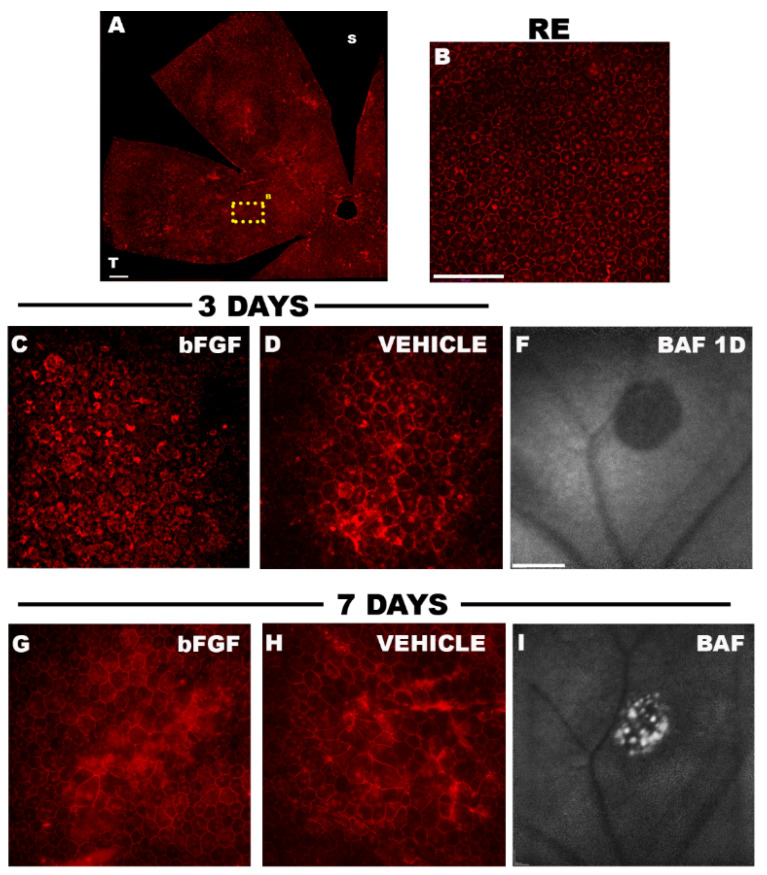
RPE analysis ex vivo by labelling zonulas occludens (**A**–**D**,**G**,**H**) and in vivo by blue autofluorescence (BAF; **F**,**I**). In the right eyes (RE) RPE showed a hexagonal and regular morphology (**A**,**B**). Intravitreal bFGF-treated retinas (**C**) showed less pleomorphism at 3 days after LIP than vehicle retinas (treated intravitreally with saline; **D**). However, these differences disappeared 7 days after LIP with similar RPE morphology in the bFGF group and vehicles (**G**,**H**). In addition, RPE alterations were observed by BAF one day after LIP with a hypo-autofluorescent circle in the lesion area leading to a hyper-autofluorescent spot in the circular lesion area at 7 days (**F**,**I**). Bar scale in (**A**) = 250 µm; in (**B**–**D**,**G**,**H**) = 100 µ; in (**F**,**I**) = 600 µm.

The RPE and photoreceptors are closely linked and form a functional unit, so that the death of one leads to secondary degeneration of the other [89]. Numerous studies have documented both in vivo and in vitro that photo-exposure causes the release of ROS and cytokines in the RPE leading to structural and functional alteration and death of the RPE and thus to the disruption of the outer BRB [25,32,43,90,91,92]. To study the integrity of the RPE and to determine its involvement in phototoxic injury and whether RPE injury is a cause or a consequence of photoreceptor degeneration, the integrity and morphology of the RPE has been studied both in vivo and ex vivo in the blue light focal phototoxicity model (Figure 2) [40]. In vivo study of RPE with the blue autofluorescence filter (BAF) showed a circular hypo-autofluorescent area 24 h after LIP that progressively changed to a hyper-autofluorescent lesion circumscribed to the lesion area (Figure 2). The natural autofluorescence of the RPE described by many authors in phototoxicity, visible with the BAF filter of the SD-OCT, is a consequence of the accumulation of lipofuscin granules in the RPE [93,94,95,96,97]. The hypo-autofluorescent pattern during the first study times (1 and 3 days after LIP) could be due to a screening effect produced by the hyper-refringent reaction visualised on SD-OCT and localised in the outer layer the day after LIP [98]. Circumscribed hyper-autofluorescent mottling in the area of the lesion in the upper temporal quadrant reveals signs of structural alteration of the RPE and increased metabolism and accumulation of lipofuscin in the RPE. The ex vivo study showed a significant morphological alteration restricted to the lesion area. Figure 2A,B shows the morphology of the non-photo-exposed control-right RPE cells forming a mosaic of mono- or binucleated hexagonal cells. At 3 days after LIP, the morphology of these cells shows a structural alteration, even more pronounced at 7 days, as shown in panels D and H of Figure 2 of vehicle-treated retinas (saline injection) after LIP induction. These morphological changes consisted of increased pleomorphism, evident at 3 days after LIP, and a reduction in cell density that was significant at 7 days.

According to the results observed in the in vivo study by BAF, SD-OCT and the ex vivo study by immunohistochemistry, where morphological alterations are observed in the RPE and a significant decrease in the thickness of the outer retina at 3 days measured by SD-OCT and a significant decrease in the number of outer segments of cones in the area of the lesion that progressively advances up to 7 days after LIP. Consequently, RPE damage is simultaneous with photoreceptor damage after LIP and as a functional unit they degenerate in parallel.

## 3. Neuroprotection in Focal Phototoxicity Models

Previous studies have documented the reproducibility and reliability of this blue LED focal phototoxic lesion model for testing different neuroprotective substances [37,38,39,40]. These studies have described the effect of these substances on the resident cone population in the lesion area, located in the superior temporal quadrant of the retina. The neuroprotective agents tested have been neurotrophic factors [37,38,39,40], the selective alpha-2 agonist (Brimonidine) [37,39] and minocycline, a tetracycline antibiotic aimed at reducing retinal microglia activation and inhibiting caspases activation [40].

### 3.1. Neurotrophic Factors

Neurotrophic factors are molecules that promote the survival, growth and differentiation of neurons. Although most of them are peptides, there are numerous neuroprotective molecules of different nature. Both in models of phototoxicity [37,38,39] and in models of photoreceptor degeneration [99], numerous molecules have been tested to prevent or delay photoreceptor death [100]. Basic fibroblastic growth factor (bFGF), brain derived neurotrophic factor (BDNF), ciliary neurotrophic factor (CNTF) and pigment epithelium derived factor (PEDF) have been tested in the focal blue LIP model administered intravitreally just after LIP induction.

#### 3.1.1. Basic Fibroblastic Growth Factor (bFGF)

Although its function is not yet fully understood, this factor increases glycogen synthase kinase and cyclic AMP-binding protein phosphorylation [101]. bFGF has been shown to be effective for photoreceptor survival both in models of inherited photoreceptors [99,102] and in models of diffuse white light phototoxicity [103]. 

The neuroprotective effect of bFGF has been demonstrated in the focal phototoxicity model in albino rats [37] as well as in albino [38,39] and pigmented mice [40]. Intravitreal injection of bFGF (0.5 μg, 27 pmol) just after LIP resulted in an increased number of cone population in the area of the phototoxic lesion at 7 days compared to retinas that were treated with vehicle. Specifically, there was 46% rescue of S-cones and 38% rescue of L-cones in PCAs of albino rat retinas [37] and 30–40% rescue of S-cones in PCAs of albino mouse retinas [38,39]. A slightly lower rescue of arrestin^+^ cones was documented in PCAs of pigmented mouse retinas, reaching 25% [40]. In addition, retinas treated with intravitreal bFGF showed a minor reduction in retinal thickness (in vivo), at the expense of the outer retina, at 5 days after LIP, although this effect was transient and at 7 days the bFGF groups showed similar values to the control groups. Although an alteration of RPE cells after blue light photo-exposure had previously been characterised [43,52,92], Miralles de Imperial-Ollero et al. (2021) [40] showed for the first time the effect of exogenous bFGF after focal LIP (Figure 2). Intravitreal administration of bFGF did not show a neuroprotective effect on the retinal RPE in the area of the phototoxic lesion at the different time points of the study (Figure 2). However, the mean cell area of the RPE cells of bFGF-treated eyes showed a similar mean cell area to that of the RPE of the right control eyes at 3 days post-LIP. Therefore, the preservation of cell morphology at 3 days of LIP may be related to the neuroprotective effect of bFGF observed in the immunohistochemical study of the arrestin^+^ cones at 7 days of LIP.

#### 3.1.2. Brain Derived Neurotrophic Factor (BDNF)

BDNF, a member of the neurotrophin family, is a neuroprotective factor produced by neurons and glia throughout the central nervous system (CNS) that plays an important role in neuronal maturation, neuronal synapses and plasticity [104]. BDNF has been shown to improve the survival of retinal cells after different lesions, such as RGCs after axotomy [105,106,107,108,109] or ocular hypertension [110,111]. In phototoxicity models, BDNF has been shown to enhance photoreceptor survival against light damage in both diffuse [112,113,114] and focal [37,38,39] light phototoxicity models. 

Intravitreal administration of BDNF (2.5 μg, 92 pmol) just after light exposure in models of focal phototoxicity had a neuroprotective effect on the S- and L- opsin population in the lesion area at 7 days after LIP in both rat and albino mice with rescue of 46% of S-cones and 31% of L-cones within the PCAs of albino rat retinas [37] and 35–49% of S-cones within the PCAs of albino mouse retinas [38,39] compared to the vehicle group. 

#### 3.1.3. Ciliary Neurotrophic Factor (CNTF)

Expressed by Müller cells, CNTF is an extracellular signaling protein in the neuroretina and interphotoreceptor matrix, which associates with the membranes of the RPE, Müller and photoreceptor cells [115,116,117]. CNTF is one of the most studied retinal trophic factors. Numerous studies have shown a protective effect of intravitreally administered CNTF on RGCs after optic nerve section [106,118,119] and, concerning the outer retina, CNTF has been shown to be effective in preventing excessive retinal 11-cis production to protect cones and rods from phototoxic damage [116], and its neuroprotective effect has been documented in models of hereditary degeneration [99,116,120]. 

However, the CNTF has shown discrepant results in the focal LIP model. A single intravitreal injection of CNTF (0.4 μg, 18 pmol) just after induction of focal LIP in albino rats did not show significant results in the number of S- and L-cones within PCAs 7 days after LIP [37]. However, half of CNTF (0.2 μg, 9 pmol) injected intravitreally into the eyes of photo-exposed albino mice did have a positive result on the rescue of S-cones within PCAs, recovering 47%, a result similar to the other trophic factors analysed [39]. These disparate results suggest that overdose of CNTF is ineffective in cone rescue after induction of phototoxicity.

#### 3.1.4. Pigment Epithelium Derived Factor (PEDF)

PEDF is a very potent neuroprotective agent that has been shown to be effective against glutamate excitotoxicity and oxidative damage [121,122,123,124,125,126]. PEDF has been shown to be effective for photoreceptor survival in both models of inherited degeneration [127,128] and models of diffuse photo-exposure photoreceptor degeneration [129]. 

Intravitreal PEDF (2 μg, 10 pmol) has also been shown to be effective in protecting against focal LIP in rats [37] and albino mice [38]. PEDF-treated albino rat PCAs had 60% and 29% rescue of S- and L-cones, respectively, and 30% rescue of S-cones in albino mice. However, it was demonstrated that lower doses of PEDF (2 or 6 pmol) were not effective in rescuing S-cones at the lesion site in albino mice and showed no significant difference compared to those treated with vehicle [38]. In addition, to study the active protein fragments of PEDF in S-cone rescue, PEDF 17-mer, 17-mer[H105A] or 17-mer[R99A] mutants (all at 10 pmol), injected just after LIP, were studied. The results of this study showed that cone survival effects were mediated by interactions between the 17-mer region of the PEDF molecule, more specifically with the 17-mer[H105A] mutant and its receptor PEDF-R [38]. This had been previously documented in vitro [130] but this was the first in vivo study to support this [38].

### 3.2. Brimonidine (BMD)

Brimonidine (BMD) is an α-2 adrenergic agonist drug widely used for the treatment of ocular hypertension and glaucoma [131,132]. In addition to its ability to reduce intraocular pressure, this drug has been shown to improve the survival of RGCs in models of retinal ischemia [133,134,135,136,137], in animal models of glaucoma [138,139,140,141] and in the survival of photoreceptors in animal models of diabetes [142] and in models of diffuse phototoxicity [143]. 

In addition, the effect of topical administration of brimonidine (two drops (2.5 μL) of 1% BMD in 0.9% NaCl administered three times a day) on the focal blue LIP model has been studied. Its effect has been studied by starting to administer the drug just on the day of LIP induction or the day before in both rats [37] and albino mice [39]. The results showed that there was no significant difference between administering the drug on the same day of LIP induction or the day before and its administration had a positive effect on cone rescue within the PCA, with 49–52% rescue of S-cones and 14–17% rescue of L-cones in albino rats [37] and 36–46% rescue of S-cones in albino mice [39].

### 3.3. Minocycline

Minocycline is an antibiotic of the tetracycline family widely known in neuroprotection studies for its inhibition of retinal and CNS microglia and inhibition of caspases activation [99,144,145,146,147,148,149]. In addition, it has been shown to be effective in neuroprotection in models of RGCs injury [150,151,152,153,154,155] or in both models of hereditary degeneration [99,156] and in models of diffuse photoreceptor degeneration due to photo-exposure [145,157].

However, minocycline administration had no positive effect on S-cone rescue in the pigmented mouse model of LIP [40]. Following previous studies [99,148], the protocol of intraperitoneal administration of minocycline (45 mg/mL) was started the day before LIP induction and injected daily thereafter. In addition, it was also ineffective in stopping or slowing phototoxic degeneration in retinal thickness measured in vivo by SD-OCT. Moreover, the possible synergistic effect of co-administration of minocycline in combination with bFGF, which had been shown to be effective in cone rescue in the same LIP model, was studied. However, in contrast to two other models of inherited photoreceptor degeneration in RCS and P23H rats [99], this drug combination did not enhance the rescue of cones within PCAs that was rescued by single administration of bFGF. Therefore, retinal inflammation does not appear to be a determining factor in photoreceptor degeneration after LIP, as is the case in other models of photoreceptor degeneration in which minocycline has been shown to be effective [99,145,148,157]. The lack of efficacy of minocycline as a neuroprotectant in this model could be due to the fact that the injury is caused by a high accumulation of energy in the form of blue light, which is highly toxic to the retina [23], directly leading to the death of photoreceptors by apoptosis [22,23,39]. This hypothesis could be supported by the findings of other authors in other models of photoreceptor degeneration in which microglial activation and migration occurs after the onset of photoreceptor degeneration [77]. LIP induction resulted in increased activation of Iba-1^+^ cells in the outer retinal layers of the lesion area, which was increased in those retinas treated with bFGF or its vehicle by intravitreal injection. This microglial activation following intravitreal injection has been previously documented by other authors [158]. In this case, intraperitoneal administration of minocycline significantly reduced the number of active Iba-1^+^ cells located in the lesion area compared to retinas treated with a single intravitreal injection of bFGF or vehicle at 7 days after LIP [40]. This effect of minocycline on the Iba-1^+^ cell population is similar to those already described by other authors in previous studies [99,148].

## 4. Limitations of the Model

Focal LIP is a reliable and reproducible model of phototoxicity that produces a small lesion in the superior-temporal quadrant of the retina that resembles macular degeneration. However, the small size of the lesion makes quantitative analysis of the effect of phototoxicity on the cone population more difficult. Therefore, these studies require restricting the study areas to pre-fixed sized circles centred on the centre of the lesion. This allows for detailed studies of the effect of focal damage on retinal cell populations and to test the efficacy of possible neuroprotective therapies.

Another aspect is that the focal phototoxicity model has only been tested in female rats and mice. The continued use of females is due to the fact that males and females may have different sized eyeballs [83], which may vary the convergence of light rays in the retina. Future studies should adapt this model to male rodents to study possible differences in retinal phototoxicity due to the sex of the animal. In addition, since the age and weight of the rodents affect the population density of photoreceptors in the retina due to retinal growth [83], the weight and age of the animals must be controlled and limited especially in this model. Changes in the size of the rat or mouse eye could affect the size of the lesion as well as the interpretation of photoreceptor degeneration.

In albino mice, focal LIP has only been studied in S-cones. These cones represent a high percentage of the total number of cones in the albino mouse retina (dual and generic S-cones); however, future studies should study the L-cone population in this strain [39]. Furthermore, the RPE study has only been performed in pigmented mice and in the centre of the lesion [40]. Future studies should analyse the effect of phototoxicity in albino rodents to study the importance of visual pigment in RPE degeneration and extend the study to more peripheral areas of the lesion.

## 5. Concluding Remarks

The blue LED-induced focal phototoxic injury model has demonstrated to be reliable and reproducible in several rodent strains. This lesion mainly affects the outer retina, which progressively reduces its thickness as the cones and the RPE are affected. In parallel to this process, there is an activation of cells of microglial cells surrounding the lesion area. In addition, this model has been feasible to test different neuroprotective strategies that can help to understand and act against the onset of AMD.

## Figures and Tables

**Table 1:** Published studies using diffuse LED phototoxicity.

Author	Rodent Strain	Wavelength	Time Exposure	Intensity	Time of Study
Shang et al., 2014 [26]	Sprague-Dawley rats	White and blue (460 nm)	On/Off cycles of 12 h for 3, 9 or 28 days	750 lux	After LED exposure
Jaadane et al., 2015 [54]	Wistar rats	White, blue (449, 467, 473 nm) and blue-green (507 nm)	Constant for 6, 12, 18, 24, 48 or 72 h	White: 2680 cd/m^2^; Blue: 102, 234, 268 cd/m^2^; Blue-Green 643 cd/m^2^	After LED exposure
Jaadane et al., 2017 [32]	Wistar rats	White	Constant for 4, 5, 6 12, 18 or 24 h	White: 2680 cd/m^2^	After LED exposure
Krigel et al., 2016 [23]	Wistar and Long Evans rats	White, blue (460 ± 5 nm) and green (530 ± 10 nm)	Constant for acute exposure: 24 h	Acute exposure: White: 500, 1000, 1500, 6000 lux Blue and green: 500 lux	7 days
			On/Off cycles of 12 h for long-term exposure: 8 or 28 days	500 lux	After LED exposure
Lin et al., 2017 [51]	Brown-Norway rats	Blue (460 nm)	Periodic cycles from 30 min to 3 h per day for 28 days	150 lux	After LED exposure
Kim et al., 2016 [36]	BALB/c mice	Blue (460 ± 10 nm)	Constant for 2 h	Illuminance-dependent (ID) groups: 1000, 2000, 3000, 6000 lux; Time-dependent (TD) group: 2000 lux	ID groups: 5 days. TD groups: 24, 48, 72 h
Nakamura et al., 2017 [42]	ddY mice	Blue (456 nm)	Constant for 2 h	400 and 800 lux	5 days
Nakamura et al., 2018 [43]	C57BL/6 mice	Blue (456 nm)	Constant for 3 h for 3 consecutive days	1100 lux	7 days
Wielgus et al., 2010 [27]	Sprague-Dawley rats	Blue (450 nm)	Constant for 6 h	750 lux	After LED exposure

Published studies of diffuse LED phototoxicity in rodents. The table specifies the rodent strain, type of LED used, time and intensity of exposure and time points of study.

## Data Availability

The data presented in this study are available on request from the corresponding author.
